# Electrochemical Detection of Dopamine using a Phenyl Carboxylic Acid‐Functionalized Electrode Made by Electrochemical Reduction of a Diazonium Salt

**DOI:** 10.1002/open.202200233

**Published:** 2022-12-07

**Authors:** Saima Anjum, Baohua Lou, Amiza Tahir, Aziz‐ur‐Rehman, M. Rehan H. Shah Gilani, Guobao Xu

**Affiliations:** ^1^ State Key Laboratory of Electroanalytical Chemistry Changchun Institute of Applied Chemistry Chinese Academy of Sciences Changchun Jilin 130022 P.R. China; ^2^ Chinese Academy of Sciences University of Chinese Academy of Sciences No. 19A Yuquanlu Beijing 100049 P.R. China; ^3^ Department of Chemistry Govt. Sadiq College Women University Bahawalpur Pakistan; ^4^ Department of Chemistry Baghdad-ul-Jadeed Campus The Islamia University of Bahawalpur Bahawalpur Pakistan; ^5^ Institute of Chemical Sciences Bahauddin Zakariya University Multan 60800 Pakistan; ^6^ University of Science and Technology of China Anhui 230026 P.R. China

**Keywords:** ascorbic acid, chemically modified electrode, diazonium, dopamine analysis, electrochemical sensing

## Abstract

A glassy carbon electrode (GCE) has been modified by an in situ electrochemical reduction of an aryldiazonium salt generated from the reaction of 4‐aminobenzoic acid and sodium nitrite in acidic ethanolic solution. The as‐prepared phenyl carboxylic acid‐modified glassy carbon electrode has been, for the first time, used for the electrochemical determination of dopamine. Under optimal experimental parameters, outstanding electrocatalytic activity, high sensitivity at a LOD of 5.6×10^−9^ m, and broad linearity of 0.1 to 1000 μm were obtained. The crafted electrochemical platform demonstrated excellent stability, specificity, and anti‐interference capability towards the sensing of dopamine.

## Graphical abstract

**A robust phenyl carboxylic acid‐modified electrode** has been prepared by electrochemical reduction of a diazonium salt. The surface of the modified electrode is negatively charged, enabling not only sensitive detection of dopamine but also excellent selectivity in the presence of ascorbic acid.

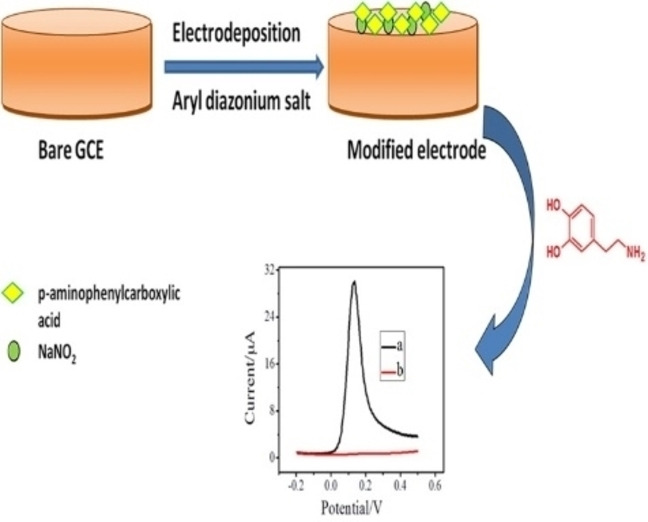

## Introduction

The process of neurotransmission is performed by neurotransmitter and it is also vital in the development and persistence of addiction.^[1]^ Dopamine (DA), a neurotransmitter, is a key catecholamine that is easily oxidized making it suitable for electroanalysis. DA is present in high amounts in a region of the brain called the caudate nucleus and the variations in its concentrations in the extracellular brain fluids can be sensed or measured via modified voltammetric microelectrodes.^[2]^ DA detection requires high sensitivity due to the metabolic actions of DA that occurs at micro‐level concentrations as well as high selectivity/specificity owing to the presence of other electroactive species (such as ascorbic acid [AA]) in the extracellular fluids that are generally present in much higher concentrations than DA itself and can inversely interact with the sensor.^[3]^ Therefore, in advance studies, the modified electrochemical sensing platforms are extensively investigated owing to their advantageous characteristics including high efficiency, cost‐effectiveness, eco‐friendly, convenient, rapid, and precise nature for the determination of DA that is a typical indicator of regular functioning.^[4]^ Furthermore, brain reward circuitry underlying addiction also comprises the dopaminergic neurons in VTA.^[5]^ Fast‐scan CV at micro‐electrodes of carbon‐fiber is an ultra‐sensitive platform with high selectivity rendering it an excellent methodology for the determination of the oxidizable neurotransmitters together with DA at cellular and tissue levels.^[6]^ Amine accumulation causes an elevation in the anionic sites on the sensing surface that assists its protonation at physiological pH as DA. However, overoxidation lessens the selectivity of the electrode and higher response time of the developed sensor towards DA. Savéant et al. investigated an approach to functionalize carbon surfaces employing aryl radicals grafting that are synthesized via electrochemical reduction of diazonium salts.^[7]^ The electrochemical reduction method has been accredited as an adaptable approach to functionalize carbon‐electrode surface.^[8]^ By considering the efficient role of DA in various body functions and as a neurotransmitter,^[9]^ the development of a robust and advanced approach for DA investigation becomes an absolute necessity. Differential pulse voltammetry/DPV confers sensitive analysis of neurotransmitters but being time‐consuming DPV analysis is not preferred. Owing to the exceptional characteristics and fast scan rate together with high sensitivity and robust analysis, square wave voltammetry is favored over other available analytical methods for the sensitive detection of DA.^[10]^ Studies are being executed to establish a novel methodology for sensitive detection of DA via chemically modified electrodes which confer good sensitivity, excellent selectivity, reproducibility, and stability.^[11]^

In present investigation, we modified glassy carbon electrode (GCE) by the electrochemical reduction of 4‐carboxyphenyldiazonium generated from the reaction of 4‐aminobenzoic acid with nitrous acid to make phenyl carboxylic acid‐modified GCE (PC‐GCE). The PC‐GCE has been explored for DA detection for the first time. Its application for selective determination of DA in the presence of AA was investigated.

## Results and Discussion

### Preparation of PC‐GCE and its mechanism

**Scheme 1:**
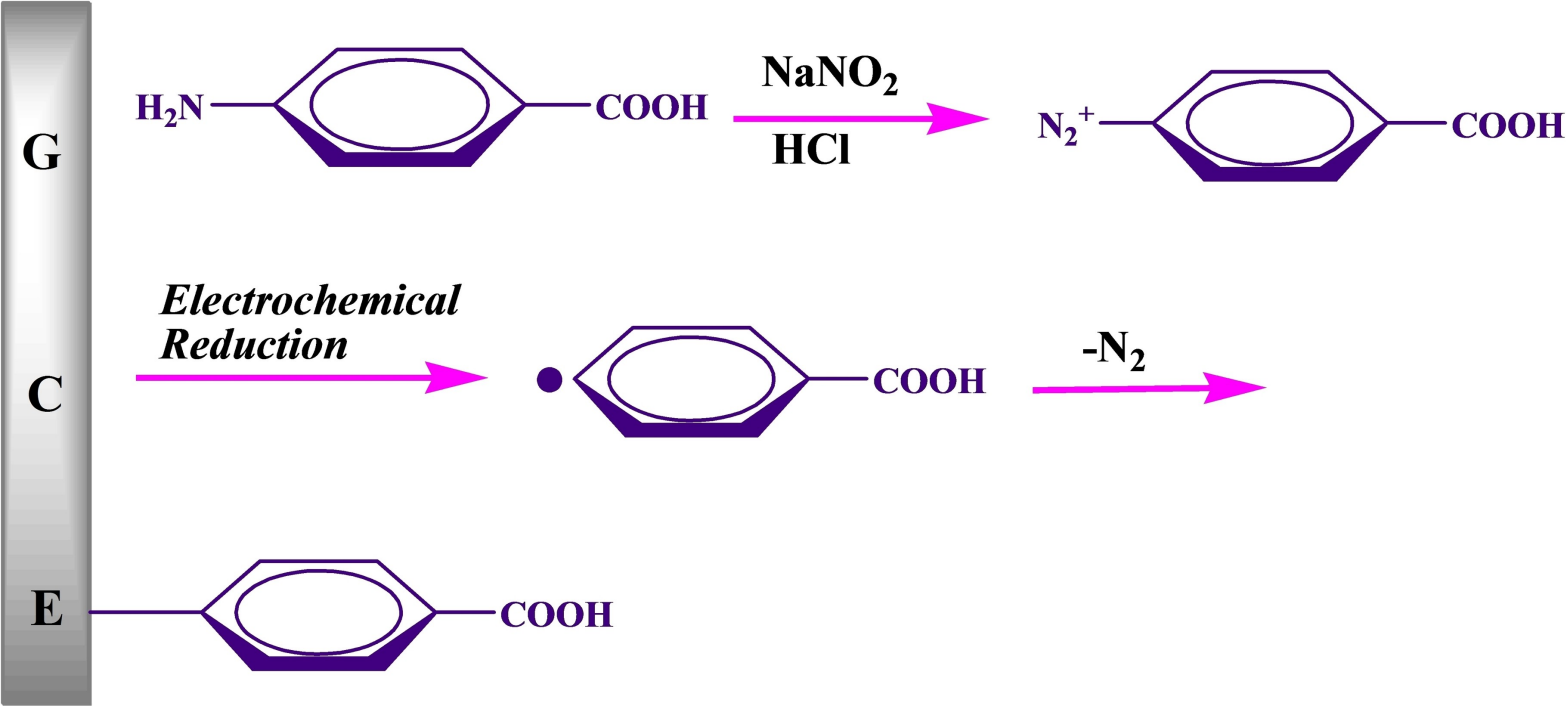
Schematic illustration of the modification mechanism of PC‐GCE.

The GCE was modified by aryldiazonium salts obtained by the reduction of 4‐aminobenzoic acid in the presence of nitrous acid. The mechanism of surface modification of GCE is as follows. Firstly, 4‐aminobenzoic acid reacted with nitrous acid to produce 4‐carboxyphenyldiazonium. Then, the electrochemical reduction of electrogenerated 4‐carboxyphenyldiazonium produced phenyl carboxylic acid radicals on the surface of GCE. Finally, these phenyl carboxylic acid radicals reacted with glassy carbon to make PC‐GCE (Scheme 1).

### Characteristics of PC‐GCE films

**Figure 1:**
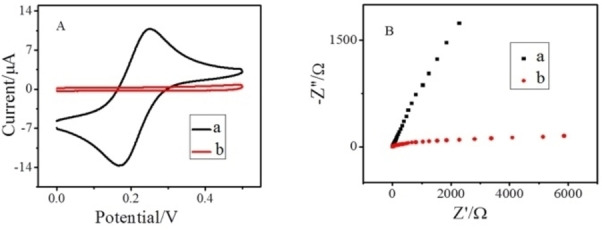
A) Cyclic voltammograms of 5 mm K_3_[Fe(CN)_6_] on (a) GCE and (b) PC‐GCE. B) Nyquist plots of FTEIS of 5 mm K_3_[Fe(CN)_6_] on (a) GCE and (b) PC‐GCE.

The designated electrode was compared with bare GCE to scrutinize the electrochemical characteristics of the designated PC‐GCE. Figure 1A shows the cyclic voltammetry behavior of bare GCE and the PC‐GCE recorded in 5 mm [Fe(CN)_6_]^3−/4−^. A typical redox signal of Fe(CN)_6_^3−/4−^ was observed on the bare GCE. Contrarily, the electrochemical signal of [Fe(CN)_6_]^3−/4−^ was completely restrained on the PC‐GCE, which could be accredited to the presence of electrostatic repulsive forces between phenyl carboxylic group and [Fe(CN)_6_]^3−/4−^ redox couple and the blocking effect of the film.^[12]^ To further ensure the electrochemical activity, the EIS responses of bare GCE and PC‐GCE in 5 mm [Fe(CN)_6_]^3−/4−^ were also examined as shown in Figure 1B. In the Nyquist plot, the semicircle domain of PC‐GCE is wider in comparison to that of GCE reflecting the electron transfer resistance which is consistent with the cyclic voltammetry analysis.^[12]^

### Electrochemical behavior of DA and AA at the designated PC‐GCE

**Figure 2:**
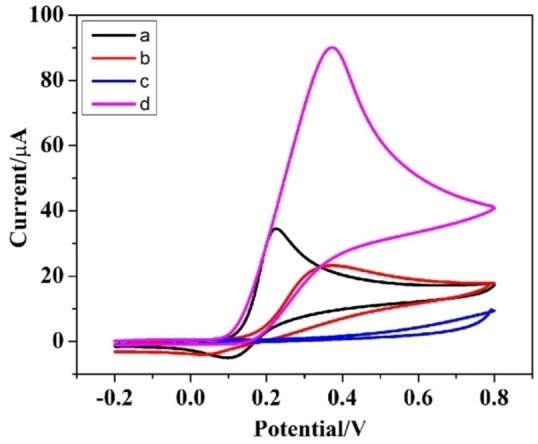
Cyclic voltammograms of (a) DA on PC‐GCE, (b) DA on bare GCE, (c) AA on PC‐GCE, and (d) AA on bare GCE.

Figure 2 illustrates the CV behavior of bare GCE and PC‐GCE in 10 mm AA and 1 mm DA in 0.1 m PBS (pH 7.0). All the CV measurements demonstrate quasi‐reversible peaks of bare and developed GCE. On modified electrode/PC‐GCE, a sharp oxidation peak corresponding to DA was observed whereas no redox peak corresponding to ascorbic acid was observed demonstrating the minimal oxidation of AA, whilst the DA oxidation was enhanced at the PC‐GCE.^[12]^ At pH 7.0, the deprotonation of the carboxyl group on PC‐GCE makes the surface of PC‐GCE negatively charged. The electrostatic attraction between negatively charged electrode surface and positively charged DA facilitates the electron transfer of DA. The electrostatic repulsion between negatively charged electrode surface and negatively charged AA inhibits the electron transfer of AA. Hence, the decorated electrode/PC‐GCE can be effectively employed for selective probing of DA even in the presence of AA.

**Figure 3:**
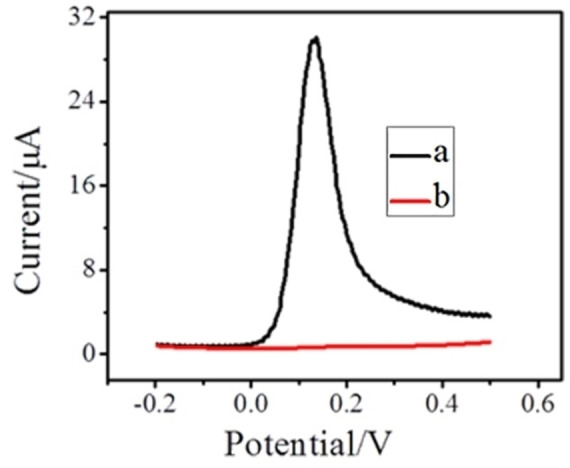
Square wave voltammograms in (a) 1 mm DA and (b) 10 mm AA on PC‐GCE.

Figure 3 depicts SWVs of 10 mm AA and 1 mm DA at decorated PC‐GCE in 0.1 m PBS (pH 7.0). DA exhibits a strong peak while AA shows a negligible peak at PC‐GCE, indicating good selectivity. The selectivity of the modified electrode is ascribed to −COOH groups that are crafted at the surface of designated probe encompassing the attachment of the positively charged DA while resisting the adsorption of negatively charged AA molecules on the modified electrode at pH 7.0.^[4a]^

### Effect of pH, deposition time, and frequency on oxidation of DA

**Figure 4:**
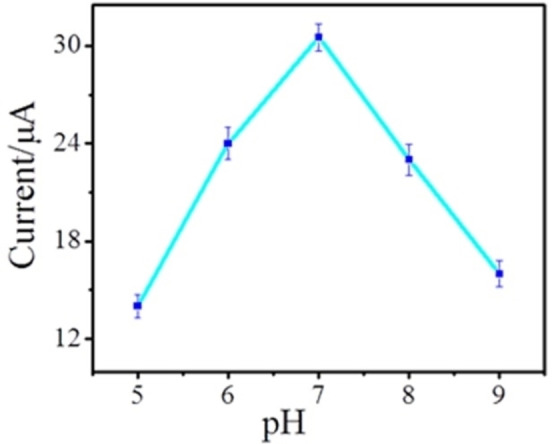
Effect of pH of solution on amperometric steady‐state current response in a 0.1 m PBS containing 1.0 mm DA on PC‐GCE

For the selection of a suitable pH to enhance the signal of the designated sensor, the SWVs of 1 mm DA in 0.1 m PBS of diversified pH at the decorated PC‐GCE were recorded as illustrated in Figure 4. The maximum peak response of DA was obtained at pH 7.0 which exhibited a decline with further increase in pH. This behavior can be assigned to the deprotonation of −COOH functional groups that are deposited on the electrode by increasing solution pH, hence, increasing the attachment of DA at the designated electrode. The SWV peak current was gradually elevated from pH 5.0–7.0. The decline in the peak response with increasing pH can be ascribed to the decreased protonation degree of DA.^[13]^ Beyond pH 7.0, the number of −COOH groups on modified GCE was increased whereas the DA carrying positive charge decreased which result in weak electrostatic attractions between COO^−^ and DA moieties. Owing to the decrease in peak current of DA with the increase in pH in the range of 7.0–9.0, pH 7.0 was selected as the optimum pH value for further analysis.

**Figure 5:**
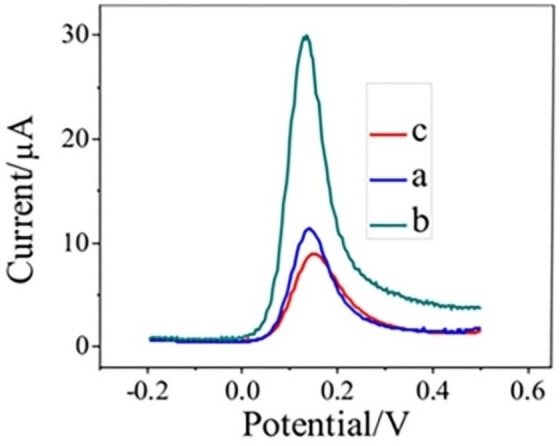
Square wave voltammograms of 1 mm DA on PC‐GCE at the frequency of (a) 10 Hz, (b) 15 Hz, and (c) 20 Hz.

**Figure 6:**
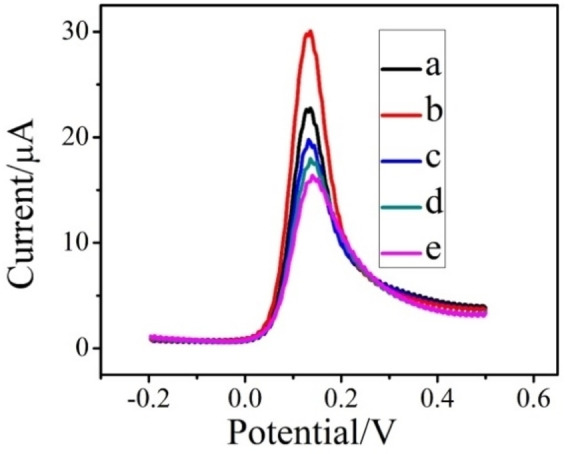
Square wave voltammograms of 1 mm DA on PC‐GCE at deposition time 15 s (a), 30 s (b), 60 s (c), 90 s (d) and 120 s (e).

Figures 5 and 6 depict the effect of frequency and deposition time (Deposition time shows the duration allotted for the attachment of analyte on the surface of the modified electrode in the accumulation step) on the peak response of DA. As exhibited in Figure 5, the highest peak current was acquired at a frequency of 15 Hz which was further employed as an optimal condition in all proceedings measurements. The effect of deposition time on the peak current of DA under optimum pH at the PC‐GCE is given in Figure 6. It was obvious that the peak current increased by increasing the deposition time from 15 s to 30 s in SWV and then decreased from 60 s to onwards (120 s and 240 s). From Figure 6, it was observed that maximum current was obtained at deposition time 30 s. Thus, a deposition time of 30 s was chosen for further analyses.

### Effect of modification time on oxidation of DA

**Figure 7:**
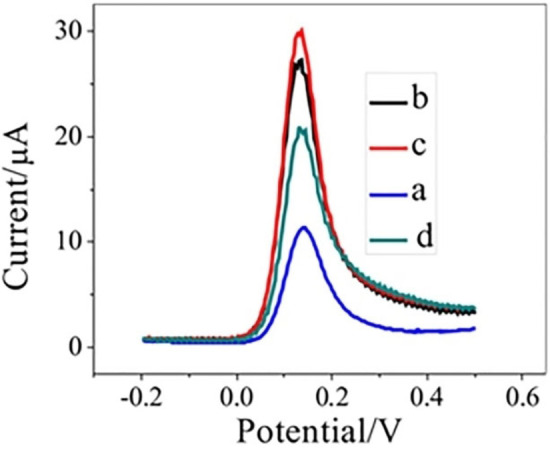
Square wave voltammograms of 1 mm DA on PC‐GCE at the modification time of (a) 60 sec, (b) 120 sec, (c) 240 sec, and (d) 480 sec.

Under optimal parameters, the influence of modification time (Modification time is the time which is given for the deposition of sensing material on the surface of electrode which in turn can enhance surface area and electrochemical properties of electrode) on the peak responses of DA at the decorated PC‐GCE is represented in Figure 7. The resulting peaks reveal growth in the current response by increasing the modification time. The highest peak response was recorded at a modification time of 240 s whereas a decrease in the response current was observed by further increasing modification time. Therefore, the modification time of 240 s was fixed as an optimum modification time for further investigations.

### Determination of DA

**Figure 8:**
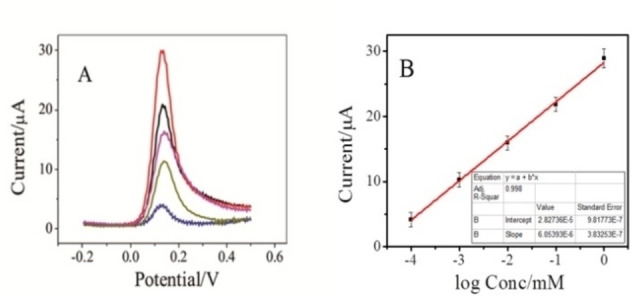
A) Square wave voltammograms of various concentrations of DA 10^−3^, 10^−4^, 10^−5^, 10^−6,^ and 10^−7^ m in 0.1 m PBS (pH 7) on PC‐GCE. Scan rate, 100 mV s^−1^. B) Calibration curve between current and various concentrations of DA 10^−3^, 10^−4^, 10^−5^, 10^−6^ and 10^−7^ m in 0.1 m PBS (pH 7), scan rate, 100 mV s^−1^.

Under the optimum parameters, the SWVs of PC‐GCE were scrutinized for different concentrations of DA in 0.1 m PBS as depicted in Figure 8A. As illustrated in Figure 8B, a linear enhance in the stripping current response is attained in the range of 0.1 to 1000 μm with an R^2^ of 0.998. The LOD was estimated to be 5.6×10^−9^ m at a S/N of 3. We have listed a comparison of this modified electrode with other previously reported electrodes in Table 1. The designated electrode proved to possess a lower detection limit with significant linear range and sensitivity than most of the reported electrodes.^[14]^ The reproducibility and stability of the developed electrode were examined by repetitive cyclic voltammetric measurements in 1 mm DA in 0.1 m PBS of pH 7. The recorded RSD of the proposed methodology was estimated to be 3.1 % after 50 consecutive CVs, signifying that the decorated probe had a remarkable reproducibility with an effective degree of accuracy and precision. The modified sensing platform was retained for 15 days in the air to investigate the stability of the designated platform. The recorded response showed that 92.9 % of the initial current was retained.

**Table 1:** Comparison of efficiency of electrochemical methods for DA determination.

Modifier for GCE	Linear Range	Limit of Detection	Reference
ZnO/Al_2_O_3_ nanocomposite	5×10^−6^–7×10^−4^ m	2.0×10^−6^ m	[15]
β‐Cyclodextrin (β‐CD) / Se‐doped carbon quantum dots (Se‐CQDs)	60–1000 μm	0.02 μm	[16]
Acid‐functionalized multi‐wall carbon nanotubes	3–200 μm	0.8 μm	[17]
Pre‐anodized helical multi‐walled carbon nanotubes	1×10^−7^–2×10^−6^ m and 6×10^−5^–2×10^−4^ m	2.1×10^−8^ m	[18]
CdTe QDs	1–400 μm	0.3 μm	[20]
Poly(2‐(*N*‐morpholine) ethane sulfonic acid)/RGO modified electrode	1.0 μm–30 μm (30 μm–100 μm), 0.05 μm–100 μm and 0.1 μm–100 μm	0.43 μm, 0.0062 μm and 0.056 μm	[19]
Hydrogenating carbon electrodes	1 mm–1 μm	0.14–0.15 μm	[20]
MIP decorated CNTs	5.0×10^−11^–5.0×10^−6^ m	1.0×10^−11^ m	[21]
Nano‐MoS_2_‐modified gold electrode	1.0×10^−11^ m to 1.0×10^−5^ m	2.3×10^−12^ m	[22]
*p*‐Aminophenyl carboxylic acid and NaNO_2_	1×10^−3^–1×10^−7^ m	5.6×10^−9^ m	Present work

### Interference study

**Figure 9:**
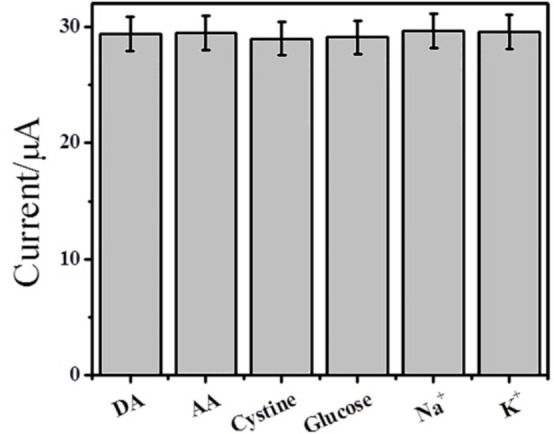
Comparison of the current intensity of 1×10^−3^ M DA and current intensity of 1×10^−3^ M DA with the addition of 1 mm AA, glucose, and cystine, 1000‐fold Na^+^ and K^+^. Error bars show the relative standard deviation of three replicate determinations.

To examine the influence of interfering species on the prepared GC electrode, its peak responses were explored by analyzing the solution comprising 1 mm DA which was added with a 100‐fold higher concentration of citric acid, glucose, and cystine, together with a 1000‐fold higher concentration of Na^+^ and K^+^. As depicted in Figure 9, the common interferents caused no obvious changes to recorded amperometric responses even in the presence of 100‐folds of physiological interfering species indicating that −COOH hindered the diffusion of interfering agents. Therefore, the procedure can be efficiently employed for the analysis of DA in real samples.

## Conclusion

The proposed sensing strategy combines high sensitivity and excellent anti‐sensing properties towards ascorbic acid (AA) and other electro‐active groups. These characteristic features make the phenyl carboxylic acid‐modified electrode advantageous for the selective detection of dopamine (DA) in neutral pH (7.0) with an enhanced sensitivity, a remarkable LOD and a wide linear range. Additionally, the innovative method proves to be an excellent way for the selective detection of DA in real samples.

## Experimental Section

### Chemicals

DA, *p*‐amino phenyl carboxylic acid, and ascorbic acid (AA) were purchased from Sigma Aldrich. All other reagents were of analytical grade and were used as such. Double distilled water was used throughout the experiment.

### Preparation of the designated Electrode (PC‐GCE)

Firstly, the glassy carbon electrode/GCE was refined by using alumina slurry of 0.05 μm grain size followed by rinsing with deionized water. The developed electrode was sonicated in ultra‐pure distilled water and ethanol for several minutes to eliminate the remains of alumina. To prepare the modified sensor, the electrode was dried at room temperature. The polished and dried GC electrode was coated in an acidic ethanolic solution comprising 10 mm 4‐aminobenzoic acid in ethanol (1 mL), 1.0 m HCl (1 mL), and 0.1 m NaNO_2_ (0.11 mL) at ambient temperature under an applied voltage of −0.45 V.^[23]^ The coated electrode was carefully rinsed with double distilled water and ethanol resulting in phenyl carboxylic acid‐modified GCE (PC‐GCE).

### Analytical procedure for the determination of dopamine

The electrochemical studies including CV and Voltammetric analysis were conducted via CHI660A electrochemical workstation together with a three probe electrochemical cell consisting of modified GC (3.0 mm in diameter) as indicator electrode, a Pt‐wire as the auxiliary electrode, and a silver/silver chloride (3 m KCl) electrode as a reference electrode. All the electrochemical studies were scrutinized with respect to the reference electrode and all the measurements were performed at ambient temperature. The sample solution was continuously stirred during the pre‐concentration step using a stirrer. The sensor was immersed in 1.0 mm DA solution in 0.1 m PBS to maintain the pH of the solution at pH 7.0. The dependence of peak responses on modification time was analyzed in the range from 60–480 s. Furthermore, the effect of pH, accumulation potential, frequency, and deposition time on current signals of DA was scrutinized. All the electrochemical investigations including SWV and CVs at bare G.C and developed PC‐GCE of 1.0 mm DA solution were executed under fitted parameters.

The SWV analysis was examined in the potential range of −0.2 to 0.5 V, potential step height of 4 mV, pulse height of 25 mV, frequency of 15 Hz, and quiet time of 10 s. The developed methodology was determined to possess remarkable sensitivity together with excellent linearity in the range of 0.1 μm to 1000 μm. Studies were carefully conducted to further ensure the anti‐interfering traits of the sensing platform along with the stability, selectivity, and reproducibility.^[24]^

## Conflict of interest

The authors declare no conflict of interest.

1

## Supporting information

As a service to our authors and readers, this journal provides supporting information supplied by the authors. Such materials are peer reviewed and may be re‐organized for online delivery, but are not copy‐edited or typeset. Technical support issues arising from supporting information (other than missing files) should be addressed to the authors.

Supporting Information

## Data Availability

The data that support the findings of this study are available from the corresponding author upon reasonable request.
